# Postoperative pain after endodontic reintervention: a randomized clinical trial

**DOI:** 10.1590/0103-6440202204785

**Published:** 2022-06-24

**Authors:** Andressa Raquel Spohr, Samantha Rodrigues Xavier, Cristiana Pereira Malta, Tatiana Pereira-Cenci, Fernanda Geraldo Pappen, Renata Dornelles Morgental

**Affiliations:** 1 Graduate Program in Dentistry, Federal University of Pelotas(UFPel), Pelotas, RS, Brazil.; 2 Graduate Program in Dental Sciences, Federal University of Santa Maria (UFSM), Santa Maria, RS, Brazil.

**Keywords:** Pain, endodontics, root canal preparation, reintervention

## Abstract

The present randomized clinical trial compared the prevalence and intensity of postoperative pain in cases of endodontic reintervention using manual or engine-driven reciprocating instruments. As secondary objectives, the analgesic intake and time required for the root canal filling removal and re-instrumentation were also evaluated. Forty-eight individuals with an endodontically treated single-rooted tooth diagnosed with asymptomatic apical periodontitis were included in the study. Patients were randomly assigned to two comparison groups (n=24/group): reintervention with stainless steel manual instruments or a nickel-titanium reciprocating system (Reciproc; VDW, Munich, Germany). The endodontic reintervention was performed in two sessions with a calcium hydroxide-based intracanal medication applied for 14 days before root canal obturation. Working time for the root canal filling removal and re-instrumentation was recorded with a digital stopwatch. After each visit, postoperative pain intensity was assessed at 12, 24, and 48 hours and seven days using the Numerical Rating Scale (NRS). The patients were also asked about analgesic intake. Data were analyzed using Pearson chi-square, T and Mann-Whitney U tests (α=0.05). No significant differences between groups were found regarding the prevalence and intensity of pain or the need for analgesic intake at any time point (P > 0.05). Working time was significantly shorter in the reciprocating group (18 versus 41 minutes). In conclusion, manual and reciprocating instruments achieved the same results in terms of prevalence and intensity of postoperative pain and analgesic intake. However, filling material removal and re-instrumentation of the root canals were more than twice as fast when using the reciprocating system.

## Resumo

O objetivo deste ensaio clínico randomizado foi comparar a prevalência e a intensidade da dor pós-operatória em casos de retratamento endodôntico, utilizando instrumentos manuais ou reciprocantes (automatizados). O tempo necessário para desobturação e reinstrumentação do canal radicular também foi avaliado. Quarenta e oito indivíduos possuindo um dente unirradicular tratado endodonticamente e portador de periodontite apical assintomática foram incluídos no estudo. Os pacientes foram aleatoriamente distribuídos em dois grupos (n=24/grupo): retratamento com instrumentos manuais de aço inoxidável ou um sistema reciprocante de níquel-titânio (Reciproc; VDW, Munique, Alemanha). A reintervenção endodôntica foi realizada em duas consultas, sendo aplicada medicação intracanal à base de hidróxido de cálcio por 14 dias, antes da obturação. O tempo clínico gasto com os protocolos de desobturação e reinstrumentação do canal radicular foi registrado com um cronômetro digital. Após cada visita, a intensidade da dor pós-operatória foi avaliada em 12, 24, 48 horas e 7 dias por meio da escala de estimativa numérica (*Numerical Rating Scale - NRS*). Além do registro da dor, os pacientes foram questionados quanto ao uso de analgésicos. Os dados obtidos foram analisados por testes Qui-quadrado e Mann-Whitney (α=0.05). Não foi detectada diferença significativa entre os grupos quanto à prevalência e intensidade da dor ou uso de analgésicos em nenhum dos períodos avaliados. O tempo clínico foi significativamente menor no grupo reciprocante (18 versus 41 minutos). Pode-se concluir que os instrumentos manuais e reciprocantes foram equivalentes quanto à prevalência e intensidade de dor pós-operatória e uso de analgésicos, mas a desobturação e reinstrumentação do canal radicular foram duas vezes mais rápidas com o sistema reciprocante.

## Introduction

The success of endodontic therapy depends on the adequate cleaning, shaping, and filling of root canals as well as satisfactory coronal sealing ^1^^,^^2^. In cases of apical periodontitis, such actions should lead to the elimination of endodontic pathogens or at least a reduction to a level that enables periapical healing ^1^. Microorganisms remaining within the root canal system, especially in the apical segment, constitute the main reason for treatment failure ^2^.

The most commonly recommended strategies for teeth with primary endodontic treatment failure are non-surgical reintervention (retreatment), different apical surgery modalities, or extraction and replacement with a dental implant ^2^. In most cases, non-surgical reintervention is the first choice because it is the least invasive approach.

It is important to remove all root filling material during reintervention to enable subsequent cleaning and a new filling of the endodontic space ^3^. The incomplete removal of gutta-percha and sealer hinders the removal of necrotic tissues and remaining bacteria in the root canal, which can lead to the recurrence of clinical failure ^2^^,^^3^. The techniques employed to remove the root filling material include heated instruments, special burrs, stainless steel manual files, nickel-titanium (NiTi) engine-driven systems, ultrasonic tips, and solvents ^3^^,^^4^^,^^5^.

Several *in vitro* studies have investigated the effectiveness of manual and engine-driven (rotary and reciprocating) instruments in endodontic reintervention ^3^^,^^4^^,^^5^^,^^6^^,^^7^. Some have demonstrated greater efficacy in the removal of filling material with the use of NiTi rotary instruments compared to the manual technique ^4^, whereas others have demonstrated similar ^7^ or inferior ^6^ efficacy. Despite not being originally developed for cases of reintervention, reciprocating NiTi systems have also proved effective, with a similar ^7^ or better performance compared to continuous rotation and manual techniques ^5^.

Researchers agree that the complete removal of gutta-percha and sealer poses a challenge ^2^^,^^3^; in this scenario, the conflicting results found in the literature and a large number of available systems impair the decision-making process on the part of dentists regarding what technique to use. Despite the high initial cost, NiTi rotary/reciprocating instruments enable faster material removal and re-instrumentation compared to manual instruments, as demonstrated in laboratory studies ^3^. Thus, single-file reciprocating systems may be a cost-effective option with a lower learning curve for cases of reintervention ^6^^,^^8^^,^^9^.

During endodontic reintervention procedures, filling materials, irrigating solutions, dentine shavings, tissue debris, and microorganisms can be pushed to the periapical tissues, the so-called apical extrusion ^10^^,^^11^. This situation is related to undesirable outcomes, such as inflammation, postoperative pain, and delayed periapical healing process ^12^.

Postoperative pain is a relatively frequent problem among patients submitted to endodontic procedures ^12^ and has been widely studied ^8^^,^^9^^,^^13^^,^^14^^,^^15^^,^^16^. Pain results from a complex and multifactorial process ^12^, influenced by aspects related to the patient, the tooth being treated, the dentist’s skills, and the intervention modality ^12^^,^^14^. Despite this relatively common occurrence, little data are found on postoperative pain, specifically in cases of endodontic reintervention and comparing different root canal filling removal methods ^8^^,^^9^^,^^16^.

Therefore, this study aimed to investigate the prevalence and intensity of postoperative pain after endodontic reintervention (root canal filling removal and re-instrumentation) in single-rooted teeth with asymptomatic apical periodontitis, comparing manual and reciprocating instruments. As secondary objectives, the analgesic intake and the time required for the root canal filling removal and re-instrumentation were also evaluated. The null hypothesis was that both groups would be similar in terms of (a) prevalence and intensity of postoperative pain, (b) analgesic intake, and (c) working time.

## Materials and methods

### Study design

The present study is a prospective, single-center, parallel, randomized clinical trial and received approval from the local research ethics committee (certificate number: 51074515.4.0000.5318). The protocol was registered online (ClinicalTrial.gov; NCT03743233). This study was reported according to the guidelines of the CONSORT Statement (www.consort-statement.org). All eligible patients received complete information on the study’s objectives, methods, and risks. Those who agreed to participate signed an informed consent form.

### Sample size calculation

A previous study evaluating the postoperative pain rate after endodontic retreatment ^16^ was considered for sample calculation using the software Sealed EnvelopeTM (Exmouth House, London UK). An alpha-type error of 0.05 and a beta power of 0.80 were specified, considering 43% of cases presenting no postoperative pain in 12 hours, using manual instrumentation, 67% in the reciprocating group, and a non-inferiority limit 2.0. The minimal estimated sample size for each group was computed as n=23 to determine the postoperative pain differences between the experimental groups. Potential patient dropouts were considered to improve the statistical power of acquired data.

### Participant selection

Approximately 350 patients sought the Dental School of the Federal University of Pelotas (UFPel; Pelotas, RS, Brazil) with an indication for endodontic reintervention between January 2017 and December 2018. Included participants were male and female adults (>18years) requiring endodontic reintervention in an asymptomatic single-rooted tooth with a single canal and persistent/secondary apical periodontitis (Periapical Index, PAI ≥ 3). PAI was evaluated in the initial periapical radiograph by a blinded and calibrated examiner (weight kappa = 0.853).

The exclusion criteria were: use of analgesics, anti-inflammatories, or other pain-modulating drugs, systemic disease (e.g., hypertension, arthritis, and kidney disorder), pregnancy, untreated periodontal disease, abnormal tooth mobility, and teeth with an excessively wide or curved canal.

**Figure 1:**
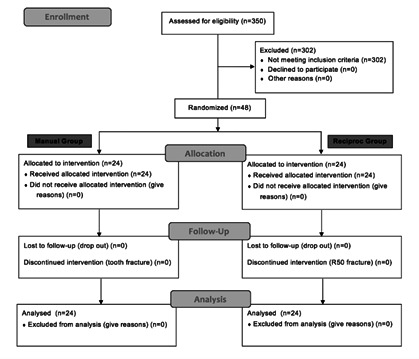
Flowchart of sample selection, treatment, and analysis following CONSORT Statement.

Individuals who met the eligibility criteria and agreed to participate in the study were randomly allocated to two comparison groups: manual or reciprocating technique (Figure 1). Stratified randomization was performed considering two operators (both dentists in the graduate program of dentistry/endodontics) who had undergone previous training for manual and reciprocating techniques. The randomization procedure was performed by an auxiliary researcher using a list of computer-generated random numbers (www.sealedenvelope.com / randomization / create a list). Opaque envelopes were used to ensure allocation concealment and were opened by an assistant only when the root filling material was going to be removed from the root canal.

### Clinical procedures

The same operator conducted each endodontic reintervention in two sessions. All patients received infiltrative anesthesia with 2% lidocaine and 1:100,000 epinephrine (Alphacaine 100; DFL, Rio de Janeiro, RJ, Brazil). Rubber dam isolation and removal of the previous coronal restorations were performed, following careful measures of aseptic control. The provisory working length (WL) was calculated from the apparent length of the tooth in the initial radiograph subtracting 1 to 2 mm.

The protocols for root canal filling removal and re-instrumentation were adapted from Dincer et al. ^17^. The initial removal of the root filling material was similar for all root canals in both groups. A size 2 Largo drill (Dentsply-Maillefer, Ballaigues, Switzerland) was used for removing the first 2 mm from the root canals, serving as a reservoir for the solvent solution. Approximately 0.1 mL of eucalyptol (Biodinâmica, Ibiporã, PR, Brazil) was placed in the canal to soften the gutta-percha for 30 seconds before the insertion of the first instrument. That was the only time the solvent was used.

Next, the reintervention technique to be used was determined (opening of the envelope). It was impossible to blind the operators and patients due to the very different kinematics of the two groups.

1. Manual reintervention: The cervical third was prepared with sizes #3 and #2 Gates-Glidden drills (Dentsply-Maillefer) in decreasing order. The crown-down technique was then performed until reaching the provisory WL, starting with a size #40 K-file (Dentsply-Maillefer) and reaching a size #25. The WL was determined using an electronic apex locator (Novapex; Forum Technologies, Rishon Le-Zion, Israel) and confirmed with a radiograph. The WL was defined as 1 mm short of the root apex and apical patency was achieved with a size #15 K-file. The root canals were re-instrumented up to a size #50 K-file apically and were flared cervically up to size #70.
2. Reciprocating reintervention: A Reciproc R25 (VDW) was used for removing the filling material until the provisory WL was reached. The instrument was inserted into the canal, and the electric motor (Silver; VDW) was activated, followed by the use of back-and-forth movements with a range of 3 mm. Gentle apical pressure was applied, combined with friction against the lateral walls, following the manufacturer’s instructions. WL determination was performed using electronic and radiographic methods as described above, with a size #25 K-file. Apical patency was established with a size #15 K-file. The root canal was then prepared using the Reciproc R50 instrument (VDW). After three back-and-forth movements, the instrument was removed from the canal and cleaned with sterile gauze. This procedure was repeated until reaching the WL. As Reciproc is a single-use instrument, only one tooth was prepared with each instrument.

During root canal filling removal and re-instrumentation protocols, irrigation was carried out with 2.5% sodium hypochlorite solution (NaOCl; Asfer, São Caetano do Sul, SP, Brazil) in both groups. After instrument change in the manual group or after 3‒4 pecking motions in the reciprocating group, 2 mL irrigant was used ^17^. All teeth received a total volume of 20 mL NaOCl. To achieve apical patency, a size #15 K-file was used with balanced force movement. After chemomechanical preparation, a new radiograph was taken without the insertion of instruments to analyze the possible presence of remaining filling material within the root canal. If the radiograph revealed the presence of material, the respective technique (manual or reciprocating) was repeated with the final instruments. H-files with the circumferential filing of the root canal walls ^18^ or ultrasonic tips ^4^ were also used, accompanied by abundant irrigation with NaOCl to remove the remaining material.

After these procedures, the root canals were flooded with 5 mL 17% EDTA (Biodinâmica, Ibiporã, PR, Brazil) for five minutes. Then, 5 mL NaOCl was used as the final irrigant. The canals were dried with size #50 absorbent paper tips (Dentsply, Petrópolis, RJ, Brazil) and filled with a calcium hydroxide-based intracanal medication (Calen-PMCC; S. S. White, Rio de Janeiro, RJ) for 14 days, as described previously ^19^. The teeth were temporarily sealed with glass ionomer cement (Vidrion R; S. S. White, Rio de Janeiro, RJ, Brazil).

In the second session, the same root canal filling technique was performed for all teeth in both groups. After local anesthesia and rubber dam isolation, the intracanal medication was removed. The root canal was irrigated with NaOCl and prepared with the master apical file. Before obturation, the smear layer removal was performed with EDTA, as described above, and the canal was dried with absorbent paper points (Dentsply). The gutta-percha master point was tested for tug-back at the WL and confirmed radiographically. The root canals were filled with gutta-percha points and AH Plus sealer (Dentsply DeTrey, Konstanz, Germany), using the cold lateral condensation technique. Excess gutta-percha and sealer were removed with heated condensers and a cotton pellet soaked in alcohol. A new provisory coronal restoration was performed with glass ionomer cement (Vidrion R; S. S. White, Rio de Janeiro, RJ, Brazil), followed by the final radiograph. An occlusal adjustment was performed in all teeth after each session. Finally, the patient was referred for definitive restoration at the university clinics.

At the end of each session, the participants received a pain questionnaire and a prescription for ibuprofen (400 mg every 6 hours) in pain cases for which they judged it necessary to take an analgesic drug ^8^^,^^13^. If intense pain persisted after taking the prescribed medication, the patient was instructed to contact the dentist for a reintervention or to obtain another prescription, depending on the case.

### Working time assessment

The time required for the root canal filling removal and re-instrumentation was recorded (in seconds) using a digital stopwatch (Timex T5K; Technos, Manaus, AM, Brazil). The device was activated upon placement of the solvent until the radiographic odontometry (T1) and from the onset of re-instrumentation to intracanal dressing application (T2). The total time (T1 + T2) was then calculated.

### Postoperative pain evaluation

Postoperative pain was assessed using an eleven-point numerical rating scale (NRS). Each patient received verbal and written explanations of the scale at the end of the sessions. NRS ranges from 0 (absence of pain) to 10 (worst pain imaginable), as described by Farrar et al. ^20^ and validated by Ferreira-Valente et al. ^21^. Pain intensity was measured at 12, 24, and 48 hours as well as seven days after the two sessions. Any need for analgesics and the number of pills taken were recorded. The patients were contacted by telephone at convenient and prescheduled times. An auxiliary researcher blinded to the technique used by the operator during the reintervention procedures performed this contact. With the previous approval of the participants, contacts in social media, residential addresses, and electronic addresses were maintained to avoid loss of contact with the patients.

### Data analysis

Demographic and clinical characteristics of the patients in the two groups were compared using Pearson Chi-square, T and Mann-Whitney U tests. The frequency of root canal filling extrusion after endodontic reintervention was also recorded in both groups and compared (Pearson Chi-square test). As the Shapiro-Wilk test demonstrated non-normal distribution, data on postoperative pain and working time were analyzed using the Mann-Whitney U test. Statistical significance was set at P<0.05, and the IBM SPSS Statistics v. 20 software (SPSS Inc., Chicago, IL, USA) was used for data analysis.

## Results

Table 1 displays the demographic and clinical characteristics of the patients in the manual and reciprocating groups. Both groups were similar in terms of sex, age, tooth group, dental arch, PAI index and apical extrusion of the root filling material (P > 0.05).

**Figure 2:**
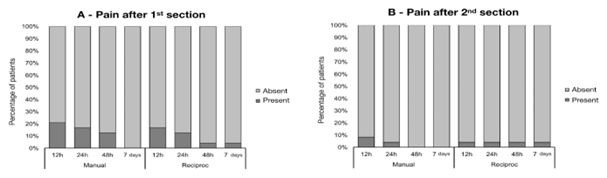
Prevalence of pain in manual and reciprocating groups at different evaluation times after first (A) and second (B) sessions.

After the first session (root canal filling removal and re-instrumentation), the prevalence of pain was low and diminished over time. Nine (18.75%) of the 48 patients reported pain at 12 h, seven (14.58%) at 24 h, four (8.33%) at 48 h, and only one (2.08%) at seven days. After the second session (endodontic obturation), three (6.25%) of the 48 patients reported pain at 12 h, two (4.16%) at 24 h, and only one (2.08%) at 48 h and seven days. No significant difference between manual and reciprocating groups was found at any time point (P = 0.296 to 1.000, Chi-square test) (Figure 2).

The intensity of postoperative pain (mean pain scores on the NRS) are displayed in Table 2. Again, no significant differences between groups were found at any time point (P = 0.282 to 1.000, Mann-Whitney test).

**Table 1:** Demographic and clinical characteristics of patients in each group.

		Manual groupn (%)(n=24)	Reciprocating group n (%)(n=24)	P
Sex	Female	20 (83.33)	16 (66.66)	0.182^*^
	Male	4 (16.67)	8 (33.34)	
Age (Mean ± SD)		46.25 ± 11.77	51.79 ± 10.89	0.097^**^
Tooth group	Incisor	17 (70.83)	14 (58.33)	0.612^*^
	Canine	2 (8.33)	2 (8.33)	
	Pre-molar	5 (20.84)	8 (33.34)	
Dental arch	Maxillary	16 (66.66)	16 (66.66)	1.000^*^
	Mandibular	8 (33.34)	8 (33.34)	
PAI [Median (Min-Max)]	4 (3-5)	3 (3-5)	0.073^***^
Root canal filling extrusion	6 (25)	3 (12.5)	0.267^*^

PAI: Periapical Index. SD: Standard deviation. ^*^ Chi-square test. ^**^ T-test. ^***^ Mann-Whitney test.

Analgesics intake was infrequent in this study. Only two patients (8.33%) in the manual group and one (4.16%) in the reciprocating group felt the need to take the medication, with no significant difference between groups (P = 0.551, Chi-square test). Regarding the number of analgesics taken, the mean was higher in the manual instrumentation (1.33 ± 5.73) compared to the reciprocating system (0.04 ± 0.20), but the difference was non-significant (P = 0.523, Mann-Whitney test).

A significant difference between groups was found regarding working time (P = 0.002, Mann-Whitney test). The filling material removal and re-instrumentation protocol were faster with the reciprocating instruments. The mean working time was 1136.17 ± 580.07 seconds (approximately 18 minutes) and 2462.54 ± 1800.05 seconds (approximately 41 minutes) in the manual and reciprocating groups, respectively.

## Discussion

The present randomized clinical trial aimed to investigate the prevalence and intensity of postoperative pain in cases of endodontic reintervention performed with stainless steel manual instruments or a NiTi reciprocating system (Reciproc; VDW). Additionally, the study evaluated the analgesic intake and the working time required for the root canal filling removal and re-instrumentation. The null hypothesis was partially rejected, as there was no significant difference between groups regarding pain and analgesic intake, but working time was significantly shorter when using the reciprocating technique. This last finding seems obvious since we compared multiple manual instruments and a single-file reciprocating system, but it seems interesting to know how faster the engine-driven system would perform.

The postoperative pain results differ from those described in two previous studies ^9^^,^^16^, which reported higher levels of pain with the manual technique in comparison to the reciprocating technique in cases of reintervention. Some researchers state that manual files cause greater extrusion of debris during the removal of the filling material than rotary/reciprocating systems ^10^, which could explain the greater pain experience. However, all instrumentation techniques are reported to cause some degree of apical extrusion ^11^^,^^17^. Findings from different studies on this issue are contradictory and some report no difference between manual and rotary/reciprocating techniques ^11^.

**Table 2:** Intensity of postoperative pain (Mean, SD, median, IQR, Min, Max) in manual and reciprocating groups at different evaluation times.

Group		Manual	Reciprocating	P*
1^st^ session				
12h	Mean (SD)	0.46 (1.14)	0.25 (0.60)	0,671
	Median	0	0	
	IQR	0	0	
	Min	0	0	
	Max	5	2	
24h	Mean (SD)	0.50 (1.31)	0.17 (0.48)	0,603
	Median	0	0	
	IQR	0	0	
	Min	0	0	
	Max	5	2	
48h	Mean (SD)	0.58 (1.97)	0.04 (0.20)	0,282
	Median	0	0	
	IQR	0	0	
	Min	0	0	
	Max	9	1	
7 days	Mean (SD)	0.00 (0.00)	0.08 (0.40)	0,317
	Median	0	0	
	IQR	0	0	
	Min	0	0	
	Max	0	2	
2^nd^ session				
12h	Mean (SD)	0.08 ± 0.28	0.04 ± 0.20	0,555
	Median	0	0	
	IQR	0	0	
	Min	0	0	
	Max	1	1	
24h	Mean (SD)	0.04 ± 0.20	0.04 ± 0.20	1,000
	Median	0	0	
	IQR	0	0	
	Min	0	0	
	Max	1	1	
48h	Mean (SD)	0.00 ± 0.00	0.04 ± 0.20	0,317
	Median	0	0	
	IQR	0	0	
	Min	0	0	
	Max	0	1	
7 days	Mean (SD)	0.00 ± 0.00	0.04 ± 0.20	0,317
	Median	0	0	
	IQR	0	0	
	Min	0	0	
	Max	0	1	

SD: Standard deviation. IQR: Interquartile range. * Mann-Whitney test.

Curiously, another clinical trial found less postoperative pain with the manual technique compared to rotary/reciprocating systems ^15^. The authors used a modified step-back manual technique. In the present study, a crown-down manual technique was employed, in which the cervical and middle thirds are prepared first, largely reducing the microbial load in the root canal and consequently reducing the possibility of driving contaminated debris into periapical tissues ^22^, which could result in pain.

Furthermore, the direct comparison of studies on postoperative pain is limited due to differences in the study design, preoperative status of the teeth involved, and the pain scale employed ^12^^,^^14^. The numerical rating scale (NRS) was used in the present investigation, which is widely employed in scientific studies ^13^ and has been validated in Portuguese ^21^, the native language of the participants. This scale has adequate reliability and cross-cultural adaptation as well as greater sensitivity and responsiveness compared to other pain assessment methods ^21^.

The prevalence and intensity of postoperative pain were low in the present study, which may be related to the strict intraoperative care, in which the manual and reciprocating instruments were handled with gentle movements accompanied by abundant irrigation ^8^. Another factor that may explain these findings is the selection of asymptomatic cases. A previous study demonstrated that the occurrence of postoperative pain was significantly lower when the teeth did not have a prior history of pain ^12^. Moreover, the standardization of the sample with the selection of only single-rooted teeth made the procedures easier and diminished the likelihood of complications.

As occurred with the pain outcome and as its consequence, the use of analgesics or the number of pills taken were very low, and no significant differences between groups were found. Ibuprofen was selected for the present study, as non-steroidal anti-inflammatory drugs are recommended as the first-choice medication to manage postoperative pain following endodontic treatment ^23^. A recent systematic review on the Cochrane platform found evidence supporting the use of ibuprofen as a safe, effective analgesic with few side effects when used for acute postoperative pain in adults ^24^. Moreover, ibuprofen is the most widely cited analgesic in studies addressing the effects of instrumentation techniques on postoperative pain following endodontic procedures ^8^^,^^9^.

As expected, the time required for filling material removal and re-instrumentation of the root canal was shorter when using the Reciproc system in comparison to manual instrumentation. *In vitro* studies have obtained similar results ^6^^,^^17^. In the present investigation, mean working time was reduced by more than half with the single-file reciprocating system. The cost of this system has become more affordable in recent years, and this significant reduction in clinical time can make it a viable alternative for both public and private dental services, even in developing countries such as Brazil. Other advantages of reciprocating systems include the high cutting capacity, excellent centralization of the preparation when employed in the curved canals, and the low learning curve ^7^^,^^8^^,^^13^.

Some methodological characteristics of the present study should be considered, and care must be taken when extrapolating the results to routine clinical practice. In this study, the reintervention was performed in two sessions with the insertion of a calcium hydroxide-based paste as intracanal medication. Olcay et al. ^19^ demonstrated that reintervention performed in multiple sessions was effective and detected a high success rate (85.1%), which was influenced by the size of the preexisting periapical lesion. In a recent systematic review and meta-analysis, Nunes et al. ^25^ reported that both reintervention modalities (single or multiple sessions) could be considered adequate for clinical practice, with a similar occurrence of postoperative pain. Therefore, endodontists should consider their experience and individual characteristics of the patient when choosing the best treatment approach ^25^.

One of the limitations of studies addressing pain is the subjective assessment of the patients and its multifactor nature ^14^. Postoperative pain can be influenced by aspects related to the patient and the tooth involved ^12^^,^^15^. In the present study, randomization ensured that the demographic and clinical characteristics of the patients were similar in the two comparison groups (Table 1). That is an important aspect, as sex, age, tooth group, and dental arch have already been pointed out as factors that can influence the occurrence of pain in endodontics ^12^^,^^14^. The frequency of extruded filling material is another variable that might impact postoperative pain results. This frequency was relatively low in this study and was equally distributed between the groups, although a slightly higher number was identified in the manual technique. Overfilling is not likely associated with unfavorable treatment prognosis ^2^, corroborating the observation that the root canal filling extrusion did not influence the present study.

The strength of the present study resides in its design. This randomized clinical trial followed all guidelines of the CONSORT Statement to ensure a transparent and precise report. However, the results cannot be generalized to all clinical cases and should be analyzed with caution. Therefore, the effect of instrumentation techniques on the incidence and intensity of postoperative pain should be assessed thoroughly.

In conclusion, manual and reciprocating instruments achieved the same results regarding the prevalence and intensity of postoperative pain and analgesic intake. However, filling material removal and re-instrumentation of the root canal was faster when using the reciprocating system.
